# Prevention of Advanced Cancer by Vitamin D_3_ Supplementation: Interaction by Body Mass Index Revisited

**DOI:** 10.3390/nu13051408

**Published:** 2021-04-22

**Authors:** Hermann Brenner, Sabine Kuznia, Clarissa Laetsch, Tobias Niedermaier, Ben Schöttker

**Affiliations:** 1 Division of Clinical Epidemiology and Aging Research, German Cancer Research Center (DKFZ), 69120 Heidelberg, Germany; s.kuznia@dkfz.de (S.K.); c.laetsch@dkfz.de (C.L.); t.niedermaier@dkfz.de (T.N.); b.schoettker@dkfz.de (B.S.); 2 Division of Preventive Oncology, German Cancer Research Center (DKFZ) and National Center for Tumor Diseases (NCT), 69120 Heidelberg, Germany; 3 German Cancer Consortium (DKTK), German Cancer Research Center (DKFZ), 69120 Heidelberg, Germany; 4 Network Aging Research (NAR), Heidelberg University, 69115 Heidelberg, Germany; 5 Medical Faculty Heidelberg, Heidelberg University, 69120 Heidelberg, Germany

**Keywords:** body mass index, cancer, prevention, supplementation, vitamin D

## Abstract

Meta-analyses of randomized controlled trials (RCTs) have demonstrated a protective effect of vitamin D_3_ (cholecalciferol) supplementation against cancer mortality. In the VITAL study, a RCT including 25,871 men ≥ 50 years and women ≥ 55 years, protective effects of vitamin D_3_ supplementation (2000 IU/day over a median of 5.3 years) with respect to incidence of any cancer and of advanced cancer (metastatic cancer or cancer death) were seen for normal-weight participants but not for overweight or obese participants. We aimed to explore potential reasons for this apparent variation of vitamin D effects by body mass index. We conducted complementary analyses of published data from the VITAL study on the association of body weight with cancer outcomes, stratified by vitamin D_3_ supplementation. Significantly increased risks of any cancer and of advanced cancer were seen among normal-weight participants compared to obese participants in the control group (relative risk (RR), 1.27; 95% confidence interval (CI), 1.07–1.52, and RR, 1.44; 95% CI, 1.04–1.97, respectively). No such patterns were seen in the intervention group. Among those with incident cancer, vitamin D_3_ supplementation was associated with a significantly reduced risk of advanced cancer (RR, 0.86; 95% CI, 0.74–0.99). The observed patterns point to pre-diagnostic weight loss of cancer patients and preventive effects of vitamin D_3_ supplementation from cancer progression as plausible explanations for the body mass index (BMI)—intervention interactions. Further research, including RCTs more comprehensively exploring the potential of adjuvant vitamin D therapy for cancer patients, should be pursued with priority.

## 1. Introduction

Meta-analyses of randomized controlled trials (RCTs) have demonstrated a protective effect of vitamin D_3_ (cholecalciferol) supplementation against cancer mortality [1,2,3], although the association had not reached statistical significance in the single studies included in the meta-analyses. However, a significant reduction of development of advanced (metastatic or fatal) cancer (hazard ratio (HR), 0.83; 95% confidence interval (CI), 0.69–0.99; *p* = 0.04) was recently reported, based on a secondary analysis, for one large RCT from the US, the VITAL study [4]. Furthermore, whereas a strong, statistically significant protective effect was seen among normal-weight participants (HR, 0.62; 95% CI, 0.45–0.86; *p* = 0.004), no such effect was seen for individuals with overweight or obesity (*p*-value for interaction =0.03). A similar effect modification had previously been reported for total cancer incidence (*p* = 0.002) in the VITAL study, with a protective effect of vitamin D_3_ supplementation in the normal-weight group (HR, 0.76; 95% CI, 0.63–0.90), but no effect among overweight (HR 1.04, 95% CI 0.90–1.21) or obese (HR 1.13, 95% CI 0.94–1.37) participants [5].

The reasons for this effect modification are uncertain. Potential explanations discussed by the authors include chance, the well-known need for higher-dose vitamin D_3_ supplementation for overweight and obese people [6], and a dynamic interplay between adiposity and immunomodulatory or inflammatory mediators, such as the differential modulation of natural killer (NK) cells in lean and overweight people. On the other hand, one could also have expected a stronger protection among overweight and obese people given the higher prevalence of insufficient vitamin D blood levels in these groups [6].

In this article, we report the results of additional analyses based on published data from the VITAL study that suggest an alternative explanation for the observed patterns, namely pre-diagnostic weight loss leading to an overrepresentation of people with undiagnosed cancer in the normal-weight group who might benefit most from vitamin D_3_ supplementation by protection from tumor progression.

## 2. Materials and Methods

### 2.1. Design Characteristics of the VITAL Study

Data were extracted or derived from publications from the VITAL study [4,5], which also provide details of the study design. Briefly, VITAL is a randomized, double-blind, placebo-controlled, 2 × 2 factorial trial conducted in the US that examines the benefits and risks of vitamin D_3_ (cholecalciferol, 2000 IU/day) and marine omega-3 fatty acids (1 g/day) for primary prevention of cancer and cardiovascular disease among men aged ≥50 and women aged ≥55. Overall, 25,871 participants (mean age 67.1 years, 51% female, 5106 African Americans) with no history of cancer (except non-melanoma skin cancer) or other major diseases were recruited throughout the US from November 2011 to March 2014. They were randomized to receive vitamin D_3_, marine omega-3 fatty acids, both active agents, or both placebos. Study medication ended on 31 December 2017, yielding a median 5.3-year (range 3.8–6.1 years) intervention period. Regular follow-up with respect to cancer incidence and mortality was conducted through patient questionnaires, medical records, and death certificates. Baseline 25-hydroxy-vitamin D (25(OH)D) levels were measured in blood samples from 16,956 participants using liquid chromatography-tandem mass spectrometry.

### 2.2. Data Extraction and Calculations

From the original main publication on vitamin D effects [5] and a more recent secondary analysis publication [4] of the VITAL study (results for the omega-3 fatty acids were reported in a separate article), we extracted the following data, overall and according to body mass index (BMI) category (<25, 25 to <30, ≥30 kg/m^2^): (i) the total numbers of participants in the intervention and control group; (ii) the numbers of participants with any incident cancer and any advanced cancer (metastatic cancer or cancer death) in the intervention and control group; (iii) the HRs for these outcomes associated with the intervention. In addition, the reported *p*-values for interaction were extracted.

From these data, we calculated relative risks (RRs) of both outcomes (any incident cancer and advanced cancer) and their 95% confidence intervals for participants with normal weight (BMI <25 kg/m^2^) and overweight (BMI 25 to <30 kg/m^2^) compared to obese participants (BMI ≥30 kg/m^2^). These calculations were conducted separately for the placebo group and the intervention group. We calculated RRs rather than HRs as only total numbers of participants (but not the person-times at risk) were reported for the various BMI categories.

Finally, we calculated RRs and their 95% CI to quantify the impact of vitamin D_3_ supplementation on being diagnosed with or developing advanced cancer among those with any incident cancer, overall and by BMI category. RRs and 95% CIs were calculated according to Altman [7].

## 3. Results

Table 1 summarizes the results on the effects of vitamin D_3_ supplementation on the incidence of any cancer and the incidence of advanced cancer (metastatic cancer or cancer death) as reported from the VITAL study [4,5]. Overall, vitamin D_3_ supplementation was associated with a lower incidence of both outcomes, but the association was weak and statistically not significant for the outcome any cancer. For both outcomes, a strong interaction by BMI was observed (*P*_interaction_ = 0.002 and 0.03, respectively), with a clear protective effect of vitamin D_3_ supplementation among normal weight participants, which was particularly strong for advanced cancer (HR, 0.62; 95% CI, 0.45–0.86). There was no effect among overweight and obese participants.

Our further analyses of these data (Table 2) yielded a significantly increased risk of both outcomes for normal-weight compared to obese participants in the placebo group (RR, 1.27; 95% CI, 1.07–1.52 for any cancer and RR, 1.44; 95% CI, 1.04–1.97 for advanced cancer). By contrast, normal-weight participants receiving vitamin D_3_ supplementation had a non-significantly lower risk of both any cancer and advanced cancer than obese participants in the intervention group (RR, 0.86; 95% CI, 0.71–1.03 and RR, 0.85; 95% CI, 0.59–1.20, respectively).

Finally, when looking at the risk of advanced cancer among those with any incident cancer (Table 3), vitamin D_3_ supplementation was associated with a significant 14% risk reduction in the entire study population (RR, 0.86; 95% CI, 0.74–0.99). Risk reduction was similar (albeit not statistically significant) within each of the BMI categories, with a slight gradient towards less risk reduction with increasing BMI.

## 4. Discussion

In this article, based on published results from the VITAL study, we demonstrate an inverse relationship between BMI and the risk of both any incident cancer and advanced cancer, with significantly increased risk among normal-weight participants compared to obese participants in the placebo group. No such pattern (and tentatively even an opposite pattern) was seen in the intervention group. Furthermore, among participants with incident cancer, vitamin D_3_ supplementation was associated with a reduced risk of advanced cancer. Such a reduced risk was seen in all BMI groups although it reached statistical significance only for the entire study population, and the strength of the risk reduction tended to decrease with increasing BMI. Taken together, these patterns point to weight loss by undiagnosed cancer at study baseline as a potential explanation for the observed BMI–intervention interactions, and to a role for vitamin D_3_ supplementation in preventing cancer progression.

The observed inverse association of BMI with cancer incidence in the placebo group on the first view appears to conflict with the well-established role of excess body weight as one of the major risk factors for many common and less common cancers [8,9]. A possible explanation for this seemingly unexpected observation could be the relatively short follow-up time of the VITAL study (median follow-up 5.3 years, range 3.8–6.1 years) which implies that all cancer diagnoses occurred within 0 to 6 years from recruitment. Most cancers take several years to become clinically evident. The sojourn time in the pre-clinical stage is several years for most common cancers (ranging from approximately 2 years for lung cancer to approximately 12 years for prostate cancer [10,11,12,13]). Therefore, a large proportion of cancers diagnosed during the follow-up of the VITAL study, especially cancers diagnosed in the early years of follow-up, likely were present but undiagnosed at the time of recruitment.

Pre-diagnostic weight loss is a well-known phenomenon for many cancers, and weight loss may in fact trigger cancer diagnoses in many instances [14,15]. Therefore, participants with preclinical undiagnosed cancer at baseline are expected to be overrepresented in the normal-weight group and underrepresented in the obese group of the VITAL study. Such shifts of undiagnosed cancer patients to the lower BMI groups may have led to the apparently increased cancer incidence in normal-weight compared to obese participants in the placebo group in our analyses.

Pre-diagnostic weight loss may, furthermore, be an indicator of more aggressive cancer and has been shown to be associated with worse cancer survival [16,17]. In addition, although high BMI is an established cancer risk factor [8,9], it is associated with lower chemotherapy toxicity [18] and better survival [16,19] among cancer patients. This may explain the even stronger increase in risk of advanced cancer seen in our analyses among normal-weight participants compared to obese participants in the placebo group.

Thus, the effects of vitamin D_3_ supplementation on incidence of any cancer, and the even stronger effects on incidence of advanced cancer in the normal-weight group could reflect both delayed transition from preclinical to clinically diagnosed cancer and delayed progression of clinically manifest cancer.

The results of the VITAL study, indicating a reduced risk of advanced cancer by vitamin D_3_ supplementation but no effect on overall cancer incidence, are consistent with the evidence from a large body of observational studies and other recent randomized trials. These studies have fairly consistently found higher 25(OH)D levels and vitamin D_3_ supplementation to be associated with lower cancer mortality [2,20] and better prognosis of cancer patients [21,22], but not with reduced cancer incidence [2,23]. It is worth noting, however, that 25(OH)D levels in the VITAL study were already rather high at baseline (mean: 30.8 ng/mL overall; 33.3, 29.5 and 26.7 ng/mL in the normal-weight, overweight and obese group, respectively) and further increased in the intervention group by approximately 12 ng/mL (with little difference between the BMI groups) within 1 year of supplementation (5). In the observational studies, a strong inverse association between 25(OH)D levels and cancer mortality was mostly seen at levels below 20 ng/mL [21,24]. These findings suggest that the benefits of vitamin D_3_ supplementation might be much stronger for people with vitamin D insufficiency and deficiency than the benefits demonstrated in RCTs among people with mostly adequate 25(OH)D levels, such as the VITAL study. Thus, even more pronounced preventive effects of vitamin D_3_ supplementation on cancer progression than reported by the VITAL study might be expected in studies focusing on cancer patients with 25(OH)D levels <20 ng/mL. Vitamin D insufficiency and deficiency are common among cancer patients [21], and preliminary results of randomized trials of vitamin D_3_ supplementation for cancer patients showed encouraging results, although they were mostly underpowered [25,26,27].

Our analysis has several strengths and limitations. Strengths include that the calculations are based on detailed BMI-group-specific data from the largest RCT on vitamin D_3_ supplementation among both sexes reported to date. Limitations include reliance on published data, which prohibited direct assessment of the extent and role of weight change before and after recruitment and limited the potential for taking additional covariates into account. For example, despite the randomized design of the study, the derived RRs comparing total and advanced cancer incidence between BMI groups might be confounded to some extent by uncontrolled covariates. Nevertheless, potential confounding would have been expected to affect the placebo and the intervention group to the same degree and would not explain the strongly divergent patterns seen for both groups. Furthermore, the derived RRs of vitamin D_3_ supplementation for advanced cancers among those with incident cancers should be unbiased given the randomized design and the large study size.

## 5. Conclusions

In summary, despite its limitations, our analysis supports suggestions of a major protective effect of vitamin D_3_ supplementation against cancer progression, either from pre-clinical to clinical cancer or after clinical manifestation. Our analyses also support the suggestion that the apparent interaction of vitamin D effects with BMI may result from pre-diagnostic weight loss, besides other potential mechanisms, such as the need for higher vitamin D_3_ doses to increase blood vitamin D levels in overweight and obese people. Given the proven safety and low cost of vitamin D_3_ supplementation, its use for reducing advanced cancer risk in the order of magnitude observed for the entire study population and the even stronger magnitude observed for the normal-weight study population might provide a very cost-effective approach to enhance survival perspectives of cancer patients and to reduce the toll of cancer deaths [28]. Further research including RCTs that more comprehensively examine the role of vitamin D_3_ in preventing deaths from various types of cancer should be pursued as a priority. Given the most likely favorable benefit/risk ratio, vitamin D_3_ supplementation for cancer patients and people at high cancer risk may be warranted even before results of further RCTs become available.

## Figures and Tables

**Table 1:** Incidence of any cancer and of advanced cancer according to BMI and vitamin D3 supplementation in the VITAL study (data extracted from references [4,5].)

BMI [kg/m^2^]	N Randomized		Any Incident Cancer			Advanced Incident Cancer		
			N		HR (95% CI)	N		HR (95% CI)
	VitD	Placebo	VitD	Placebo		VitD	Placebo	
Any	12,927	12,944	793	824	0.96 (0.88–1.06)	226	274	0.83 (0.69–0.99)
<25	3884	3959	206	278	0.76 (0.63–0.90)	58	96	0.62 (0.45–0.86)
25–<30	5060	5062	338	323	1.04 (0.90–1.21)	98	109	0.89 (0.68–1.17)
30+	3679	3610	228	199	1.13 (0.94–1.37)	65	61	1.05 (0.74–1.49)
*P*_interaction_					0.002			0.03

BMI, body mass index; CI, confidence interval; HR, hazard ratio; VitD, vitamin D group.

**Table 2:** Relative risks of any cancer and advanced cancer according to BMI group and vitamin D3 supplementation in the VITAL study (derived from results extracted from references [4,5] and shown in Table 1).

BMI [kg/m^2^]	Relative Risk (95% Confidence Interval)			
	Any Incident Cancer		Advanced Incident Cancer	
	VitD	Placebo	VitD	Placebo
<25	0.86 (0.71–1.03)	1.27 (1.07–1.52)	0.85 (0.59–1.20)	1.44 (1.04–1.97)
25–<30	1.08 (0.92–1.27)	1.16 (0.98–1.37)	1.10 (0.80–1.50)	1.27 (0.93–1.74)
30+	1.00 (Ref)	1.00 (Ref)	1.00 (Ref)	1.00 (Ref)

BMI, body mass index; VitD, vitamin D group.

**Table 3:** Effects of vitamin D3 supplementation on risk of advanced cancer among those with incident cancer according to BMI in the VITAL study (derived from results extracted from references [4,5] and shown in Table 1).

BMI [kg/m^2^]	Relative Risk (95% Confidence Interval)
Any	0.86 (0.74–0.99)
<25	0.82 (0.62–1.07)
25–<30	0.86 (0.69–1.08)
30+	0.93 (0.69–1.25)

BMI, body mass index.

## Data Availability

The data used in this manuscript are publicly available from previous publications and fully disclosed in Table 1 and Table 2 of the manuscript.
